# Premorbid intellectual ability in schizophrenia influence family appraisal related to cognitive impairments: a cross-sectional study on cognitive impairment and family assessments

**DOI:** 10.1186/s12888-022-03879-2

**Published:** 2022-03-31

**Authors:** Kota Ebina, Mie Matsui, Yuko Higuchi, Michio Suzuki

**Affiliations:** 1 Laboratory of Clinical Cognitive Neuroscience, Graduate School of Medical Sciences, Kanazawa University, Kanazawa, Japan; 2 Laboratory of Clinical Cognitive Neuroscience, Institute of Liberal Arts and Science, Kanazawa University, Kanazawa, 920-1192 Japan; 3 Department of Neuropsychiatry, Graduate School of Medicine and Pharmaceutical Sciences, University of Toyama, Toyama, Japan

**Keywords:** Schizophrenia, Self-assessment, Family rating, Neurocognition, Cognitive impairment, Introspective accuracy

## Abstract

**Background:**

Patients with schizophrenia are unaware of their cognitive impairments. Misperception of cognitive impairment is an important factor associated with real-world functional outcomes in patients with schizophrenia. The patient’s family member plays a crucial role in detecting patients’ cognitive impairments when the patients are unaware of their own cognitive impairments. Previous studies have reported that not only the patient’s subjective rating, but also the patient’s family members’ rating of their cognitive impairment may not be precise. However, it is unclear why family ratings are inaccurate, and which factors impact family ratings. This study investigated whether family ratings differed significantly from the patients’ subjective ratings of the patients’ cognitive impairments and sought to determine the reason for the differences between the family ratings and the patients’ neurocognitive performances. We investigated the relationship between patients’ subjective ratings, family ratings for patients’ cognitive impairments, neuropsychological performance, and other aspects, including premorbid IQ and clinical symptoms.

**Method:**

We evaluated 44 patients with schizophrenia for cognitive function using neuropsychological tests; in addition, both the patients and their families rated the patients’ cognitive impairments through questionnaires. We used the Mann–Whitney *U* test to examine whether the family ratings differed significantly from the patients’ self-reported ratings of their cognitive impairment. We conducted multiple regression analysis and structural equation modeling to determine why the patients’ subjective ratings and the family ratings were not definitively associated with the patients’ neurocognitive performances. We performed multiple regression analysis with a stepwise method with neurocognitive performance, premorbid IQ, positive symptoms, and negative symptoms as independent variables and family ratings of patients’ cognitive impairments as dependent variables.

**Results:**

We found that the family ratings differed significantly from the patients’ subjective self-reported ratings of their cognitive impairments. Our results showed that the premorbid IQ of patients is the strongest predictor of family ratings. Furthermore, among the neurocognitive domains, only the processing speed of patients was associated with family ratings.

**Conclusions:**

We found that the family ratings were not consistent with the patients’ subjective self-reported ratings and the family ratings were most affected by the patients’ premorbid intellectual abilities. These results suggest that the families’ current assessments of the patients’ current cognitive impairments were affected by the patients’ premorbid intellectual ability rather than the patients’ current neurocognitive performance. Patients’ processing speed predicted family ratings; however, family members' ratings were not related to verbal learning/memory, executive function, and language of patients. Therefore, our findings highlight that patients’ family ratings may differ from patients’ subjective ratings, results of performance-based neuropsychological tests, and clinician ratings.

## Background

Cognitive impairment, including deficits in attention, verbal learning, working memory, and executive function, is a core problem in schizophrenia [1, 2]. Patients with schizophrenia are unaware of their own cognitive impairment [3–5]. The unawareness of cognitive impairments has been defined as impairment of neurocognitive “introspective accuracy (IA)” in schizophrenia patients, and IA refers to “how well individuals evaluate their own abilities, skills, performance, and decisions” [3]. In addition, the direction of misestimation of their own abilities has been labeled introspective bias (IB) [3]. IB refers to the direction of inaccuracy of patients’ subjective evaluations. This, when patients subjectively rate their own cognitive abilities as higher than their actual cognitive abilities, that is considered overestimation (direction: positive); when patients rate their cognition as lower than their actual cognitive abilities, that is considered underestimation (direction: negative). IA is a crucial factor that contributes to a patient’s functional outcomes [6]. The greater overestimation of their own functioning is associated with impaired neurocognitive abilities and lower levels of depressive symptoms [7–9]. In a recent study of IA in the domain of social cognition, less severe depressive symptoms were associated with lower self-reported impairment in social functioning and higher social cognitive ability [10]. These results suggest that levels of depressive symptoms and cognitive performance are crucial signals determining the accuracy and direction (underestimation or overestimation) of patients’ subjective self-reported evaluations of their own cognitive impairments. Furthermore, it has been suggested that weakened executive function [11, 12] and autistic traits [13] are also determinants of IA impairment. To date, the problem of self-assessment in patients with schizophrenia has been investigated by comparing the patient's self-rating with informant-rating (high-contact clinician or case manager, or patient’s family member) or using correlations between self-rating and performance of objective neuropsychological tests [6, 8].

Early detection and treatment are important for improving outcomes in patients with schizophrenia. Patients with schizophrenia often show impaired cognitive performance, including verbal memory and executive function/working memory, since the clinical high-risk state [14]. The patients’ families, who live with and knows the patients very well, may be able to recognize the patients’ cognitive impairment in the prodromal phase. Family awareness of a patient’s cognitive impairment may be crucial for the early detection and treatment of schizophrenia. However, previous studies have reported that the rating of patients’ family members as the informant was less associated with patients’ performance on neurocognitive tests and functional capacity than ratings by a high-contact clinician [15, 16]. These previous findings suggest that the rating of family members for a patient’s cognitive ability is not always accurate. Family awareness of the patient's cognitive impairment is important for improved outcomes, although few studies have focused on the family’s assessment of the patient's cognitive impairment.

This study had two goals. One, it sought to determine whether family members’ ratings of schizophrenia patients’ cognitive impairment differed from the patient’s subjective self-reported ratings. Two, it sought to determine the reason for the differences between the family ratings and the patients’ subjective self-reported ratings of their cognitive impairments and neurocognitive performances, in particular, to identify the factors that affect family ratings.

We investigated the relationship between patients’ subjective ratings, family ratings for patients’ cognitive impairments, and neuropsychological performance using structural equation modeling (SEM). We also included patients’ premorbid IQ and positive and negative symptoms in the SEM as variables that may moderate family ratings and neurocognitive performance.

## Methods

### Participants

The participants were 44 outpatients (male = 25, female = 19) diagnosed with schizophrenia according to the ICD-10 criteria [17], treated with medication, and in stable condition. All the participants were outpatients who had participated in a previous intervention study on cognitive remediation therapy at the Department of Neuropsychiatry, Toyama University Hospital. We used the data from the previous study’s pre-intervention control group and the intervention group receiving cognitive remediation therapy. We recruited the participants from the outpatient department by displaying posters, distributing pamphlets, and contacting patients through their doctors. The patients’ diagnoses were made by psychiatrists using semistructured interviews and the diagnostic criteria in the ICD-10. The study’s inclusion criteria were as follows: (a) patients aged 20 or over; (b) patients with an ICD-10 diagnosis of schizophrenia; (c) patients with symptoms controlled by medication and not currently experiencing an acute phase; and (d) patients not currently hospitalized. The study’s exclusion criteria were as follows: (a) patients aged under 20 or over 65; (b) patients with a premorbid IQ lower than 80, as measured with the Japanese Adult Reading Test (JART) [18]; (c) patients with a history of head trauma or surgery; (d) patients with a neurological illness; and (e) patients with substance or alcohol abuse.

Written informed consent was obtained from all participants after the procedures had been fully described.

### Procedures

To evaluate the patients’ neurocognitive performance, all patients completed performance-based neuropsychological tests, including processing speed, memory, executive function, and verbal fluency. To rate the patients’ subjective and family ratings for patients’ everyday functioning, measurement of everyday cognition (ECog) [19] was performed by both patients and their family members. Clinical symptoms were evaluated using the Scale for the Assessment of Positive Symptoms (SAPS) [20] and the Scale for the Assessment of Negative Symptoms (SANS) [21]. All the neuropsychological tests were administered by psychologists trained in neuropsychological assessments. Clinical symptoms were assessed using a semistructured interview method by licensed clinical psychologists with sufficient training and clinical experience. We conducted the ECog family version with family members who were currently living with the patient and had been for a significant period, so they knew the patients well (e.g., mother, father, siblings, spouse, etc.).

### Measures

#### Assessment of clinical symptoms

We assessed the positive symptoms using the SAPS [20]. We used the composite score, which is the sum of the items “hallucinations,” “delusion,” “bizarre behavior,” and “positive formal thought disorder.” We assessed the negative symptoms using the SANS [21]. The score was the sum of global rating of “affective flattening or blunting,” “alogia,” “avolition-apathy,” “anhedonia-asociality,” and excluding "subjective complaints of emotional emptiness or loss of feeling”, “subjective rating of alogia”, “subjective complaints of avolition and apathy”, “subjective awareness of anhedonia-asociality”, and “inappropriate affect”.

Additionally, we needed to exclude the SANS global rating for “attention” and “poverty of content of speech” from the SANS composite score to match the current conceptualization of negative symptoms. However, we could not verify the scores for individual patients’ SANS items; our dataset contained only the global SANS ratings, not individual SANS item scores. Furthermore, because we took our data from a previous dataset, we had limited access to the participants’ information and could not retrospectively confirm all the SANS raw data for individual patients. Therefore, we used SANS composite scores, excluding the global rating for “attention” in the SANS. Higher scores for SAPS and SANS also indicate higher symptom severity.

#### Patient ratings and family ratings of everyday function using ECog

The ECog questionnaire assesses patients’ daily functioning [19]. This questionnaire was based on the Schizophrenia Cognition Rating Scale (SCoRS) [22, 23], which consists of 20 items related to daily living (e.g., remembering names of people you know or meet, following a television show, etc.). The SCoRS was developed to assess cognitive impairment in patients with schizophrenia and has been validated [23]. The Japanese version of the SCoRS has been shown to be valid in Japanese patients with schizophrenia [22]. The SCoRS uses a semistructured interview with patients and their informants (e.g., caregiver, family member, friend, etc.) to arrive at a global score (range: 1–10) from three perspectives: patient, informant, and rater. Hoshino and Matsui (2014) modified the SCoRS to measure the subjective cognitive impairments and family evaluations more easily by using a questionnaire without changing the items’ contents [19]. Since the method of administration and scoring differed from the official SCoRS, they called their questionnaire “ECog.” Hoshino and Matsui (2014) reported that the ECog has high internal reliability (Cronbach’s alpha = 0.93) and test–retest reliability (*r* = 0.81) in a preliminary study of the families of patients with schizophrenia [19]. This questionnaire consisted of six cognitive domains: attention, memory/learning, problem solving, working memory, language processing, and motor function. Each item is rated on a four-point scale, with higher scores reflecting a greater degree of impairment.

### Performance-based neuropsychological tests

#### Verbal learning/memory

The Japanese Verbal Learning Test (JVLT) assesses short-term verbal retention and verbal learning abilities [24]. JVLT is composed of 16 word lists (4 categories × 4 words). Participants were required to recall the word lists after the examiners had finished presenting the words. The JVLT has three trials in all, and the scores were given the sum of the number of words correctly recalled in each trial (range of scores is 0 to 48). The story recall subtest of the Japanese version of the Rivermead Behavioral Memory Test (RBMT) examines episodic memory [25, 26]. The number of sentences recalled immediately was scored (0–25).

#### Processing speed

Trail Making Test Part A (TMT-A) assesses processing speed [27]. TMT-A is a task to trace numbers in ascending order, and the numbers on the paper are arranged from 1 to 25. The time taken to trace all the numbers was scored.

### Executive function

Trail Making Test Part B (TMT-B) [27] and the Wisconsin Card Sorting Test (WCST) [28, 29] assess executive function. Participants must classify the cards given to them by the examiner and guess how to classify the cards based on the feedback of “right” or “wrong” for their classification. The number of trials and total errors required to complete the six categories were used as scores. Trail Making Tests B (TMT-B) [27] is a task of sequentially and alternately following numbers and words randomly arranged on paper in one stroke (Example 1-a-2-i-3-u-). The time taken to trace all numbers and words was scored.

### Language

The Verbal Fluency Task for the Japanese version (VFT) measures language function [30]. Participants were asked to generate as many words as possible within 60 s. We implemented five categories: words beginning with “KA”; words beginning with “TA”; ANIMALS; FRUITS; and VEGETABLES. We used the total number of words generated in each category as the score.

### Statistical analyses

We analyzed the data using IBM SPSS, Version 27 (Armonk, NY: IBM Corp.). We set the statistical significance at *p* < 0.05. We conducted the Mann–Whitney *U* test to examine whether there was a significant difference between the ECog scores of the patient and family ratings; the Shapiro–Wilk test showed that the ECog scores for the patients (*p* = 0.01) and families (*p* = 0.01) were not normally distributed. We used stepwise multiple regression analyses to investigate which variables, such as neurocognitive performance, premorbid IQ, positive symptoms, and negative symptoms, predicted ECog scores for patients and their families. We conducted SEM to investigate the relationship between family ratings, patient ratings, neurocognitive performance, premorbid IQ, and symptoms. We performed SEM using IBM SPSS AMOS, Version 27.0 (Armonk, NY: IBM Corp.).

In the first step, we generated the latent variable for neurocognitive performance from the observable variables, including TMT-A, TMT-B, VFT, RBMT memory, WCST, and JVLT. We built the model using “neurocognitive performance,” premorbid IQ, and positive and negative symptoms as predictors and the patients’ ratings and families’ ratings on ECog as the dependent variables. We calculated the latent variable “neurocognitive performance” using factor analysis in SEM using the scores, factor loadings, and errors of the individual neurocognitive tests. In other words, we calculated the latent variable (factor) “neurocognitive performance” from the scores of the observable variables TMT-A, TMT-B, VFT, RBMT memory, WCST, and JVLT using factor analysis with a one-factor structure.

In the next step, we investigated whether these predictors (i.e., “neurocognitive performance,” premorbid IQ, and positive and negative symptoms) predicted the family and patient ECog ratings. Furthermore, if the results of the SEM showed that the neurocognitive performances as a latent variable were significantly associated with the family rating of the ECog, we needed to identify which cognitive domain influenced the family rating. We also used multiple regression analysis to identify which patient’s neurocognitive domain affected the family rating of patient’s cognitive impairments, with neurocognitive performances as the independent variable.

## Results

### Demographic, clinical, neurocognitive data and distributions

Table 1 shows the means and standard deviations of the patients’ demographics and cognitive data. The mean age of the patients was 37.3 ± 11.2 (SD) years, and the mean years of education was 14.3 ± 1.8. The mean age of onset of schizophrenia was 23.7 ± 6.9 (SD) years old; the mean of illness duration was 13.6 ± 11.2 (SD) years; and the mean of antipsychotic dose (risperidone equivalent) was 5.5 ± 3.4 (SD) mg/day.

**Table 1:** Means and standard deviations of evaluations

	**Means (SD)**	**Min**	**Max**
**Demographics**			
Age (years)	37.3 (11.2)	20	62
Education (years)	14.3 (1.8)	12	20
JART	104.2 (8.8)	82	123
Age of onset (years old)	23.7 (6.9)	15	45
Illness duration (years)	13.6 (11.2)	1	43
Antipsychotic dose (risperidone equivalent) was	5.5 (3.4)	0	15.5
**Clinical symptoms**			
SAPS	20.6 (17.8)	0	74
SANS	34.7 (16.8)	8	75
**Neurocognitive tests**			
TMT-A time	37.3 (12.1)	20	68.9
TMT-B time	80.1 (31.1)	36	170
WSCT completed categories	5.6 (1.5)	0	6
WCST number of errors	20.4 (20.7)	5	96
RBMT immediate recall	11.9 (5.3)	2	22
JVLT recall total	28.9 (7.1)	17	43
VFT total score	57.6 (11.5)	30	81

*JART* Japanese Adult Reading Test, *SAPS* Scale for the Assessment of Positive Symptoms, *SANS* Scale for the Assessment of Negative Symptoms, *TMT-A* Trail Making Test—Part A, *TMT-B* Trail Making Test—Part B, *WCST* Wisconsin Card Sorting Test, *RBMT* Rivermead Behavioral Memory Test, *JVLT* Japanese Verbal Learning Test, *VFT* Verbal Fluency Task

### Comparison of current cognitive impairments in patient and family

We observed a statistically significant difference between patient rating and family rating in the ECog total score: (patient vs. family 15.9 ± 10.5 (SD) vs. 11.5 ± 10.0 (SD), *p* < 0.05). The family ratings for ECog were lower than the patient ratings (Table 2).

**Table 2:** Comparison of current cognitive impairments in patient and family

	**Means**	**SD**	**Mann–Whitney-U**	**z**	***p***
			697.50	-2.11	< 0.05
Patient rating	15.9	10.5			
Family rating	11.5	10.0			

*SD* Standard deviations, *z* z-score, *p p*-value

### The relationships between family rating, patient rating, and neurocognitive performances, premorbid IQ, and symptoms

We performed stepwise multiple regression analyses with neurocognitive performance, JART, SAPS, and SANS as independent variables, and family ratings of ECog scores as dependent variables. The results showed that JART was the strongest predictor of family ratings for ECog (β = -0.48, *p* < 0.01). The TMT-A score was the second predictor of family ratings for ECog next to JART (β = 0.32, *p* < 0.05) (Table 3).

**Table 3:** Multiple regression analysis of patients’ neurocognitive performances, premorbid IQ, and symptoms on family rating

Predictor	β	t	*p*	95% CI	Adj R^2^	F
					0.29	8.75
JART	-0.48	-3.48	< 0.01	-0.86: -0.23		
TMT-A	0.32	2.34	< 0.05	0.04: 0.50		

The results of multiple regression analysis for predicting family rating from patients’ neurocognitive performances, premorbid IQ, and symptoms. β: Standardizing Coefficient, *t* t-value, *p p*-value, *CI* Confidence Interval, *Adj R*^*2*^ Adjusted R^2^, *F* F-value, *JART* Japanese Adult Reading Test, *TMT-A* Trail Making Test—Part A

Stepwise multiple regression analyses were also performed with neurocognitive performance, JART, SAPS, and SANS as independent variables, and patient ratings of ECog scores as dependent variables. The results showed that none of the independent variables predicted the patients’ subjective rating of ECog.

### SEM of the family rating, patient rating, and neurocognitive performances

**Fig. 1:**
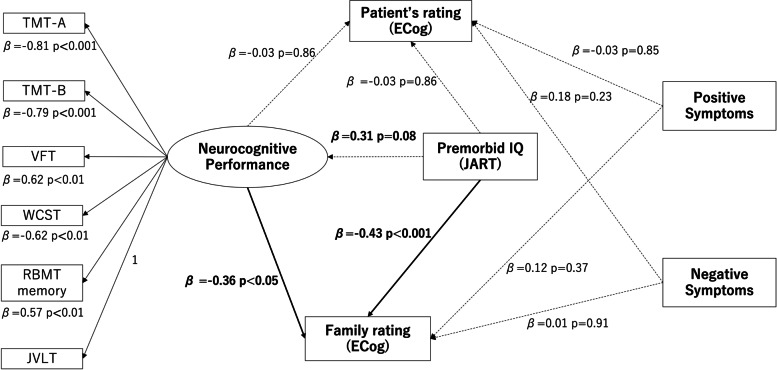
The relationship between neurocognitive performance, patient’s subjective rating, and family rating: ECog of family rating was significantly associated with patient’s neurocognitive performances and premorbid IQ. Premorbid IQ was not significantly related to neurocognitive performance. Positive symptoms and negative symptoms were not associated with family rating. Note. TMT-A: Trail Making Test—Part A. TMT-B: Trail Making Test—Part B. VFT: Verbal Fluency Task. WCST: Wisconsin Card Sorting Test. RBMT: Rivermead Behavioral Memory Test. JVLT: Japanese Verbal Learning Test. Neurocognitive Performance: Latent variables calculated from individual neurocognitive test scores. Patient’s rating (ECog): The score of patient’s subjective rating of ECog, Family rating (ECog): The score of family member rating of ECog, ECog: Measurement of Everyday Cognition. JART: Japanese version Adult Reading Test. Positive Symptom: The total score of Scale for the Assessment of Positive Symptoms. Negative Symptom: Scale for the Assessment of Negative Symptoms. β: Standardized partial regression coefficient: *p*: *p*-value

We examined a model in which neurocognitive performance, premorbid IQ, positive symptoms, and negative symptoms were predictors of the family rating and patient rating of ECog (Fig. 1). All the observed variables were significantly associated with generated latent variable: TMT-A (β = -0.81, *p* < 0.001), TMT-B (β = -0.79, *p* < 0.001), VFT (β = 0.62, *p* < 0.01), WCST (β = -0.62, *p* < 0.01), RBMT memory (β = 0.57, *p* < 0.01). The results of SEM indicated that neurocognitive performance (β = -0.36, *p* < 0.05) and JART (β = -0.43, *p* < 0.001) significantly predicted the family ratings of ECog. Positive symptoms (β = 0.12, *p* = 0.37) and negative symptoms (β = 0.01, *p* = 0.91) did not predict the family rating of ECog. None of the predictors, including positive symptoms (β = -0.03, *p* = 0.85), negative symptoms (β = 0.18, *p* = 0.23), premorbid IQ (β = -0.03, *p* = 0.86), and neurocognitive performance (β = -0.03, *p* = 0.86), predicted the patient rating of ECog. Premorbid IQ was not significantly associated with neurocognitive performance (β = 0.31, *p* = 0.08). This model had a good fit (RMSEA = 0.06, CFI = 0.92).

### The relationships between family rating and neurocognitive performances

We conducted multiple regression analysis to examine which neurocognitive domains predicted the family ratings of ECog. The results indicated that only TMT-A significantly predicted the family ratings of ECog (β = 0.28, *p* < 0.05). The other neurocognitive domains did not predict family ratings: TMT-B (β = -0.004, *p* = 0.98), VFT (β = -0.17, *p* = 0.20), WCST (β = -0.21, *p* = 0.12), RBMT (β = -0.25, *p* = 0.06), and JVLT (β = -0.17, *p* = 0.21) (Table 4).

**Table 4:** The relationships between family rating and neurocognitive performances

	TMT-A		TMT-B		VFT		WCST		RBMT		JVLT	
	β	*P*	β	*p*	β	*p*	β	*p*	β	*p*	β	*p*
Family rating	0.28	< 0.05	< 0.01	0.98	-0.17	0.20	-0.21	0.12	-0.25	0.06	-0.17	0.21

β: Standardizing Coefficient, *p p*-value, *TMT-A* Trail Making Test—Part A, *TMT-B* Trail Making Test—Part B, *VFT* Verbal Fluency Task, *WCST* Wisconsin Card Sorting Test, *RBMT* Rivermead Behavioral Memory Test, *JVLT* Japanese Verbal Learning Test, *VFT* Verbal Fluency Task

## Discussion

The purpose of this study was to determine whether patients’ neurocognitive performance, premorbid IQ, and clinical symptoms affect the family rating of patients’ cognitive impairments. It was unclear why the patient's family ratings were less related to the patients’ cognitive abilities than the clinicians’ ratings. We found that family ratings were significantly different from patients’ subjective evaluations of cognitive impairments. Additionally, our findings suggest that premorbid IQ is the strongest predictor of family ratings. To our knowledge, this is the first study to show that family ratings are affected by the patient's premorbid IQ, rather than neurocognitive performance or the severity of clinical symptoms.

Our results revealed that the family ratings of patients’ cognitive impairment were lower than patients’ subjective ratings. This finding is inconsistent with previous studies, in which patients with schizophrenia have shown a lack of awareness of their cognitive impairments [5, 7]. However, the problem of introspective bias in schizophrenia involves both overestimation and underestimation [3]. Regarding the distribution of overestimators of their cognitive function in schizophrenia, Bowie (2007) reported that 40% [7] and Sabbag (2012) reported that 60% of patients overestimated their cognitive function [31]. It is natural for the ratio of under-estimator to over-estimator to change with sampling. Furthermore, previous studies have reported that higher levels of daily living function and higher cognitive abilities are associated with overestimation of their own cognitive impairments [32]. The participants in this study were outpatients with stable clinical status, and most of them achieved all categories of the WCST. Although depression was not assessed, participants in this study may have had higher levels of depression or higher cognitive abilities associated with an overestimation of cognitive impairment. Further studies, including the assessment of depression, are required.

We found that a patient’s premorbid IQ was the strongest predictor of family ratings of patients’ cognitive impairments, and processing speed was the second predictor. Other cognitive domains, positive symptoms, and negative symptoms did not predict family ratings. These results suggest that family ratings are less related to patients’ cognitive performance and functional capacity than clinicians’ ratings. Moreover, family ratings are influenced not only by the current cognitive impairment of the patient, but also by the patient's premorbid intellectual ability. The results of the multiple regression analysis showed that processing speed was related to family ratings. A previous meta-analysis reported that processing speed, assessed by the digit symbol coding test, is more impaired compared to other specific cognitive domains such as verbal memory, executive function, and working memory [33]. Impairment of processing speed may be a central component of cognitive dysfunction in schizophrenia [34]. Therefore, family members may be able to recognize impairments in processing speed relatively easily in their daily lives.

The results of SEM revealed that patients’ premorbid IQ affected family rating. Since the patients’ family members had lived with the patients for a long time, they were influenced by their impressions of the patients’ premorbid (before their cognitive decline) level of functioning; the higher the patient’s premorbid functioning, the more likely the family was to underestimate the cognitive impairment after the onset. Furthermore, SEM results did not show that JART was associated with the patients’ neurocognitive performance. This finding is not consistent with previous studies, which showed that premorbid IQ estimated by JART is related to patients’ neurocognitive performance such as verbal memory, processing speed and verbal fluency estimated using the Brief Assessment of Cognition in Schizophrenia (BACS) [35]. The previous study suggested that premorbid intellectual ability may be a crucial factor that contributes to neurocognitive performance. It is possible that our analysis did not show a statistically significant association between premorbid IQ and patients’ neurocognitive performance because of the small sample size and low statistical power.

The patients’ subjective cognitive impairments were not related to their neurocognitive performance, premorbid IQ, or clinical symptoms. This result suggests that depressive symptoms, autism traits, and internalized stigma might determine patients’ subjective ratings of current cognitive impairments [9, 13, 36]. Further research focusing on more comprehensive aspects, including these indicators, is required.

This study had some limitations. Our sample size was relatively small in these analyses. Further research is needed to increase the sample size and to ensure our results. Previous studies reported that several factors, including depression and autism, were significantly related to IA in patients with schizophrenia [9, 13, 31]. We did not investigate the relationships between patients’ subjective evaluations and neurocognitive performance, considering the effects of these other factors. The SEM of this study did not include all variables related to the patient's subjective evaluation reported to date. Thus, it is unclear whether other aspects, such as depression and autism traits, were associated with family ratings.

Patients with a stable clinical status were included in this study. Further research is needed to determine whether the findings of our study can be applied to patients with different levels of cognitive impairment and the severity of clinical symptoms.

Although the IA of social cognition has gained attention [16], we did not focus on social cognition in this study. Recently, Pinkham et al. (2018) showed that patients with schizophrenia showed less IA-specific activation of social cognition in the right rostrolateral prefrontal cortex, which is involved in IA abilities in healthy controls [37]. Further studies should focus on to clarify what factors affect the family ratings of patients’ social cognition.

Although we should exclude the SANS global rating for “attention” “inappropriate affect (SANS item 6)” and “poverty of content of speech (SANS item 11)” from the SANS composite score to match the current conceptualization of negative symptoms [38], we could not exclude “poverty of content of speech”. This was because access to participant information was limited, and it was impossible to retrospectively confirm all SANS raw data for individual patients. Further research is needed to fully align scoring with the contemporary concept of negative symptoms. Finally, this study used cross-sectional design. Therefore, we could not examine the effects over a time course for the patients’ subjective ratings, the families’ ratings, or the patients’ neurocognitive performances. Further longitudinal studies are required to elucidate the reason for the lack of association between the family ratings, the patients’ subjective self-reported ratings, and the objective neurocognitive performances.

## Conclusion

We found that premorbid intellectual ability may be a crucial predictor of family ratings of patients’ cognitive impairments in daily life. Our data suggest that processing speed is a significant predictor of family ratings among neurocognitive functioning domains. These results emphasize that the evaluation of patients’ cognitive impairments by family members was different from the patient's subjective evaluation and neurocognitive performance.

## Data Availability

The datasets analyzed in this study are available from the corresponding author upon reasonable request.

## Abbreviations

IA: Introspective Accuracy
JART: Japanese version Adult Reading Test
SAPS: Scale for the Assessment of Positive Symptoms
SANS: Scale for the Assessment of Negative Symptoms
JVLT: Japanese Verbal Learning Test
WAIS-III: Wechsler Adult Intelligence Scale—Third Edition
TMT-A: Trail Making Test—Part A
TMT-B: Trail Making Test—Part B
WCST: Wisconsin Card Sorting Test
VFT: Verbal Fluency Task
SEM: Structural Equation Modeling

## References

[1] Gold JM (2004). Cognitive deficits as treatment targets in schizophrenia. Schizophr Res.

[2] Nuechterlein KH, Green MF, Kern RS, Baade LE, Barch DM, Cohen JD (2008). The MATRICS Consensus Cognitive Battery, Part 1: Test Selection, Reliability, and Validity. Am J Psychiatry.

[3] Silberstein J, Harvey PD (2019). Impaired introspective accuracy in schizophrenia: an independent predictor of functional outcomes. Cogn Neuropsychiatry.

[4] Burton CZ, Harvey PD, Patterson TL, Twamley EW (2016). Neurocognitive insight and objective cognitive functioning in schizophrenia. Schizophr Res.

[5] Medalia A, Thysen J (2008). Insight into neurocognitive dysfunction in schizophrenia. Schizophr Bull.

[6] Gould F, McGuire LS, Durand D, Sabbag S, Larrauri C, Patterson TL (2015). Self-assessment in schizophrenia: Accuracy of evaluation of cognition and everyday functioning. Neuropsychology.

[7] Bowie CR, Twamley EW, Anderson H, Halpern B, Patterson TL, Harvey PD (2007). Self-assessment of functional status in schizophrenia. J Psychiatr Res.

[8] Durand D, Strassnig M, Sabbag S, Gould F, Twamley EW, Patterson TL (2015). Factors influencing self-assessment of cognition and functioning in schizophrenia: Implications for treatment studies. Eur Neuropsychopharmacol.

[9] Harvey PD, Twamley EW, Pinkham AE, Depp CA, Patterson TL (2017). Depression in schizophrenia: Associations with cognition, functional capacity, everyday functioning, and self-assessment. Schizophr Bull.

[10] Harvey PD, Deckler E, Jones MT, Jarskog LF, Penn DL, Pinkham AE (2019). Depression and reduced emotional experience in schizophrenia: Correlations with self-reported and informant-rated everyday social functioning. J Exp Psychopathol.

[11] Prouteau A, Atzeni T, Tastet H, Bergua V, Destaillats JM, Verdoux H (2015). Neurocognitive insight and executive functioning in schizophrenia. Cogn Neuropsychiatry.

[12] Brekke JS, Kohrt B, Green MF (2001). Neuropsychological functioning as a moderator of the relationship between psychosocial functioning and the subjective experience of self and life in schizophrenia. Schizophr Bull.

[13] Harvey PD, Deckler E, Jones MT, Jarskog LF, Penn DL, Pinkham AE (2019). Autism symptoms, depression, and active social avoidance in schizophrenia: Association with self-reports and informant assessments of everyday functioning. J Psychiatr Res.

[14] Lencz T, Smith CW, McLaughlin D, Auther A, Nakayama E, Hovey L (2006). Generalized and Specific Neurocognitive Deficits in Prodromal Schizophrenia. Biol Psychiatry.

[15] Sabbag S, Twamley EM, Vella L, Heaton RK, Patterson TL, Harvey PD (2011). Assessing everyday functioning in schizophrenia: Not all informants seem equally informative. Schizophr Res.

[16] Silberstein J, Harvey PD (2019). Cognition, social cognition, and Self-assessment in schizophrenia: prediction of different elements of everyday functional outcomes. CNS Spectr.

[17] World Health Organization. The ICD-10 Classification of Mental and Behavioral Disorders: clinical descriptions and diagnostic guidelines. Geneve: World Health Organization; 1992.

[18] Matsuoka K, Uno M, Kasai K, Koyama K, Kim Y (2006). Estimation of premorbid IQ in individuals with Alzheimer’s disease using Japanese ideographic script (Kanji) compound words: Japanese version of National Adult Reading Test. Psychiatry Clin Neurosci.

[19] Hoshino T, Matsui M (2014). The current status of car driving behavior and daily cognitive functioning in patients schizophrenia: examination form the family survey. SEISHINIGAKU.

[20] Andreasen NC (1983). The Scale for the Assessment of Positive Symptoms (SPNS).

[21] Andreasen NC (1989). The Scale for the Assessment of Negative Symptoms (SANS): Conceptual and Theoretical Foundations. Br J Psychiatry.

[22] Kaneda Y, Kamioka Y, Sumiyoshi T, Furukori N, Ito T, Higuchi Y, Omori T (2010). The Schizophrenia Cognition Rating Scale Japanese Version (SCoRS-J). SEISHINIGAKU.

[23] Keefe RSE, Ph D, Poe M, Walker TM, Kang JW, Harvey PD (2006). The Schizophrenia Cognition Rating Scale: An Interview- Based Assessment and Its Relationship to Cognition, Real-World Functioning, and Functional Capacity. Am J Psychiatry.

[24] Matsui M, Yuuki H, Kato K, Kurachi M (2006). Impairment of memory organization in patients with schizophrenia or schizotypal disorder. J Int Neuropsychol Soc.

[25] Wilson BA, Cockburn J, Baddeley A (1986). The Rivermead Behavioral Memory Test (RMBT).

[26] Watamori S, Hara H, Miyamori T, Eto F (2002). The Rivermead Behavioral Memory Test (RMBT), Japanese Version, Chiba Test Center.

[27] Reitan RM, Wolfson D (1985). The Halstead-Reitan Neuropsychological Test Battery.

[28] Nelson HE (1976). A Modified Card Sorting Test Sensitive to Frontal Lobe Defects. Cortex.

[29] Heaton RK, Chelune GJ, Talley JK, Kay GG, Curtiss G (1993). Wisconsin Card Sorting Test manual: revised and expanded.

[30] Matsui M, Yuuki H, De Kato K, Takeuchi A, Nishiyama S, Bilker WB (2007). Schizotypal disorder and schizophrenia: A profile analysis of neuropsychological functioning in Japanese patients. J Int Neuropsychol Soc.

[31] Sabbag S, Twamley EW, Vella L, Heaton RK, Patterson TL, Harvey PD (2012). Predictors of the accuracy of self assessment of everyday functioning in people with schizophrenia. Schizophr Res.

[32] Kim SJ, Jung DU, Moon JJ, Jeon DW, Seo YS, Jung SS (2021). Relationship between disability self-awareness and cognitive and daily living function in schizophrenia. Schizophr Res Cogn.

[33] Dickinson D, Ramsey ME, Gold JM (2007). Overlooking the Obvious. Arch Gen Psychiatry.

[34] Sheffield JM, Karcher NR, Barch DM (2018). Cognitive Deficits in Psychotic Disorders: A Lifespan Perspective. Neuropsychol Rev.

[35] Akiyama K, Saito S, Saito A, Ozeki Y, Watanabe T, Fujii K (2016). Predictive value of premorbid IQ, negative symptoms, and age for cognitive and social functions in Japanese patients with schizophrenia: A study using the Japanese version of the Brief Assessment of Cognition in Schizophrenia. Psychiatry Res..

[36] Shin YJ, Joo YH, Kim JH (2016). Self-perceived cognitive deficits and their relationship with internalized stigma and quality of life in patients with schizophrenia. Neuropsychiatr Dis Treat.

[37] Pinkham AE, Klein HS, Hardaway GB, Kemp KC, Harvey PD (2018). Neural correlates of social cognitive introspective accuracy in schizophrenia. Schizophr Res.

[38] Marder SR, Kirkpatrick B (2014). Defining and measuring negative symptoms of schizophrenia in clinical trials. Eur Neuropsychopharmacol.

